# Novel Ultrasonographic Fatty Liver Indicator Can Predict Hepatitis in Children With Non-alcoholic Fatty Liver Disease

**DOI:** 10.3389/fped.2018.00416

**Published:** 2019-01-08

**Authors:** Hsien-Kuan Liu, Ming-Chun Yang, Yu-Tsun Su, Chi-Ming Tai, Yu-Feng Wei, I-Chun Lin, Ching-Chung Tsai

**Affiliations:** ^1^Department of Pediatrics, E-Da Hospital, I-Shou University, Kaohsiung, Taiwan; ^2^Department of Internal Medicine, E-Da Hospital, I-Shou University, Kaohsiung, Taiwan; ^3^Department of Pediatrics, Kaohsiung Chang Gung Memorial Hospital, Kaohsiung, Taiwan

**Keywords:** non-alcoholic fatty liver disease, non-invasive, hepatitis, children, semi-quantitative ultrasonographic fatty liver indicator

## Abstract

**Background:** Childhood non-alcoholic fatty liver disease (NAFLD) is a public health issue worldwide. To date, liver biopsy remains the gold standard for diagnosing the severity of NAFLD. However, this invasive procedure might contribute to complications. Owing to this reason, a good non-invasive tool to estimate NAFLD in children is urgently needed. We sought to investigate whether a non-invasive semi-quantitative ultrasonographic fatty liver indicator (US-FLI) can estimate NAFLD in children.

**Methods:** Children aged between 10 and 18 years were enrolled prospectively. Abdominal ultrasonography was performed by a single experienced pediatric gastroenterologist and the non-invasive semi-quantitative US-FLI score were used. Patients were diagnosed with NAFLD if they had a US-FLI score ≥2. The anthropometric measures, obesity-related biochemical results, and levels of tumor necrosis factor-α, interleukin-6, caspase-cleaved cytokeratin fragment of cytokeratin 18 (M30), and adiponectin were also checked.

**Results:** Overall, 117 children aged 10–18 years were enrolled. The anthropometric measures and obesity-related biochemical parameters (hsCRP, triglyceride, uric acid, AST, ALT, γ-GT, homeostatic model assessment insulin resistance (HOMA-IR), and M30) were significantly higher in the obesity group than in the non-obesity group (*p* < 0.05). Similarly, the US-FLI score was significantly higher in the obesity group than that in the non-obesity group (*p* < 0.001). Multiple linear regression showed that the US-FLI score was significantly associated with the waist-to-height ratio, uric acid, adiponectin, and M30 levels (all *p* < 0.05) in children with obesity. The US-FLI score ≥6 was the optimal cut-off point for predicting the hepatitis in children with NAFLD. The area under the receiver operating characteristic curve was 0.710 (95% CI: 0.572–0.847; *p* = 0.005).

**Conclusions:** The non-invasive US-FLI score can predict hepatitis in children with NAFLD without mandatory liver biopsy. Moreover, the waist-to-height ratio, uric acid, adiponectin, and M30 levels were significantly associated with US-FLI score in children with obesity.

## Introduction

Non-alcoholic fatty liver disease (NAFLD) is a spectrum of disease comprising simple steatosis, fibrosis, and liver cirrhosis, as well as finally results in chronic liver disease in the future. Non-alcoholic steatohepatitis (NASH), a severe form of NAFLD, is defined as a status in which steatosis is combined with inflammation and hepatocyte damage proved by histopathological examination (1). The incidence of NAFLD, which is closely linked to obesity, is also increasing worldwide in children (2, 3). To date, liver biopsy remains the gold-standard to determine the severity of NAFLD (4). However, complications of liver biopsy, such as bleeding and transient hypotension, and parental concerns are major obstacles to this invasive procedure (5). With the gradual increase in the incidence of this disease, it has been a challenge for pediatricians to develop a non-invasive tool to estimate NAFLD earlier in children.

Several biomarkers of NAFLD such as aspartate aminotransferase (AST), alanine aminotransferase (ALT), AST/ALT ratio, γ-glutamyltranspeptidase (γ-GT), insulin resistance (IR) have been reported (4, 6). Furthermore, inflammatory markers, such as tumor necrosis factor-alpha (TNF-α), interleukin-6 (IL-6), caspase-cleaved fragment of cytokeratin 18 (M30), and adiponectin have also been proposed to be highly correlated to NASH (7–9). To our knowledge, however, there is no biomarker or inflammatory marker that can predict NAFLD completely (10).

Imaging techniques are commonly used to evaluate patients with NAFLD (4). A non-invasive semi-quantitative ultrasonographic fatty liver indicator (US-FLI) is a newly designed score that has been found to be highly correlated with histopathological finding of NASH in adults (11). To our knowledge, US-FLI score has not yet been applied in children. Because parents are concerned about the invasiveness of liver biopsies, we sought to investigate whether US-FLI score is also a good tool to estimate NAFLD in children and whether it is highly associated with obesity-related clinical data and inflammatory biomarkers.

## Methods

### Study Design

This prospective study was performed from June 2015 to March 2016. Children aged between 10 and 18 years were voluntarily enrolled from an out-patient department in E-Da hospital, Taiwan. All the participants were interviewed to investigate systemic disease, recent infectious disease, previous surgery history, family history, medication use, and any possible exposure to hepatotoxicity agents. Alcohol consumption was confirmed by the participants and their caregivers, separately, to assure that all participants had no alcohol consumption history. Anthropometric measurements (body weight, body height, waist, and hip circumference, etc.) were recorded at the time of recruitment. Participants with hepatitis B or C, systemic disease, recent febrile disease, and a history of major surgery were excluded. The study was approved by the Institutional Review Board of E-Da Hospital (approval number: EMRP25104N) in accordance with the Declaration of Helsinki. Written informed consent was obtained from each participant and a parent/guardian of the participant in this study. All participants' information was de-identified before analysis.

### Clinical Evaluation and Biochemical Tests

Body weight, body height, waist/hip circumference, and blood pressure were measured after the participants removed their shoes and heavy clothing. Body mass index (BMI) was calculated using the following formula: BMI = weight (kg) / height^2^ (m^2^). Waist-to-hip ratio (WHR), waist-to-height ratio (WHtR) were also calculated. Venous blood samples were collected after fasting for 12 h. Hepatitis B surface antigen and anti-hepatitis C virus antibody were checked, and participants with positive results were excluded. Liver function and related biochemical marker test results (including AST, ALT, γ-GT, and alkaline phosphatase [ALK-P]), lipid profile (including total cholesterol [TC], triglyceride [TG], high density lipoprotein [HDL], and levels of low-density lipoprotein [LDL]), glycated hemoglobin [HbA1c], fasting serum glucose, uric acid, and C-peptide were checked. IR was calculated using HOMA-IR Calculator, version 2.2.3 as reported by Wallace et al. (12).

### Biomarker Assays

TNF-α, IL-6, M30, and adiponectin were measured. After blood sampling, centrifugation (3,000 rpm for 10 min; X-22R, Beckman Coulter, CA, USA) was immediately performed and serum samples isolated. The CK18 M30-Apoptosense enzyme-linked immunosorbent assay (ELISA) kit (PEVIVA, Bromma, Sweden) was used to quantify M30 levels in the serum. The adiponectin level was quantified using Human Adiponectin (Acrp30) ELISA Kit (BioLegend, Inc., CA, USA), TNF-α level using Human TNF-α ELISA Kit (BioLegend, Inc., CA, USA), and IL-6 levels using Human IL-6 ELISA Kit (BioLegend, Inc., CA, USA).

### Non-invasive Semi-quantitative US-FLI Score

Abdominal ultrasonography (USG) was performed by a single experienced pediatric gastroenterologist who was unaware of participants' biochemical data; all participants confirmed that they fasted overnight for at least 12 h. An ultrasound machine (Philips IE33, Amsterdam, Netherlands) was used with a C5-1 convex probe (transmitting 2.2 MHz and receiving 4.4 MHz). US-FLI score was calculated based on the publication reported by Ballestri et al. The score ranges from 2 to 8. The score is calculated as follows: (i) diagnosis of steatosis by the contrast between liver and kidney, (ii) the presence of ultrasound beam attenuated posteriorly, (iii) vessel blurring, (iv) gallbladder wall visualized difficultly, (v) difficult visualization of the diaphragm, and (vi) areas of focal sparing. Patients were diagnosed with NAFLD if they had a US-FLI score ≥2 (11).

### Statistical Analysis

Median (interquartile range) are presented as non-parametric continuous variables, proportions as categorical variables, and mean ± standard deviation as parametric continuous variables. The Pearson's χ^2^ test, Mann-Whitney *U*-test, and Student's *t*-test were used for comparisons between the non-obesity and obesity groups. Simple linear regression was used to analyze the relationship between each independent variable and US-FLI score as a dependent variable. Among these independent variables, we excluded the highly correlated and high homogeneous independent variables. Using the rule of per 10 sample size to one independent variable in the regression model. Statistical adjustment in multiple linear regressions then included WHtR, hsCRP, HOMA-IR, TG, LDL, and M30 in the non-obesity group, and WHtR, DBP, uric acid, HDL, adiponectin, and M30 in the obesity group. The stepwise method was used to determine whether these independent variables were significantly associated with US-FLI score.

Patients were diagnosed with hepatitis if they had elevated ALT value (>40 units per liter). Receiver operating characteristic (ROC) curves were calculated to determine the optimal threshold to discriminate NAFLD with hepatitis from NAFLD without hepatitis in children using US-FLI score. We identified the maximum value of Youden's index (sensitivity + specificity-1) and the optimal threshold value was used as the cut-off point to determine the sensitivity, specificity, positive predictive values of US-FLI score for detecting the presence of hepatitis in children with NAFLD.

All statistical tests were two-sided, conducted at a significance level of 0.05, and reported using *p*-value, and/or 95% confidence intervals (CI). All analyses were performed using the SPSS statistical software version 24 (IBM Corp., NY, USA).

## Results

Overall, 127 participants met the inclusion criteria during the 9 month study period. After excluding patients with underlying disease (Henoch-Schonlein purpura, *n* = 1; Hashimoto disease, *n* = 1; hepatitis B carrier, *n* = 1; focal seizure, *n* = 1), incomplete ultrasound survey (*n* = 5), and major operation history (bariatric surgery, *n* = 1), a total 117 participants were included in this study. The mean age was 13.29 ± 2.37 years, 82 (70.1%) were boys, and the median BMI was 23.59 kg/m^2^ (interquartile range: 8.37). Participants were divided into non-obesity and obesity groups based on criteria reported by Chen et al. (13). Demographic characteristics, anthropometric measurements, and laboratory data related to obesity are shown in Table 1. Age at the time of sampling and the levels of total cholesterol, LDL, HbA1c, TNF-α, and IL-6 did not significantly differ between the groups. The WHR, WHtR, blood pressure (systolic and diastolic), and levels of hsCRP, triglyceride, uric acid, AST, ALT, γ-GT, HOMA-IR, and M30 were significantly higher in the obesity group than in the non-obesity group (*p* < 0.001 for all). The HDL and adiponectin levels were significantly higher in the non-obesity group (both *p* < 0.001). Similarly, the US-FLI score was significantly higher in the obesity group than that in the non-obesity group (*p* < 0.001). The prevalence of obesity was higher in boys; this was similar to a previous report published in Taiwan (14).

**Table 1:** Factors associated with non-alcoholic fatty liver disease in patients based on body mass index.

**Variables**	**Non-obesity (*n* = 59)^a^**	**Obesity (*n* = 58)^a^**	***P***
Age	13.4 ± 2.3	13.2 ± 2.4	0.611
Boy	34 (57.6%)	48 (82.8%)	0.003
BH	156.5 (147.0–166.5)	163.0 (151.0–168.1)	0.129
BW	49.4 (42.6–56.8)	76.1 (63.6–88.5)	<0.001
BMI	20.6 (18.2–22.2)	29.0 (25.6–33.3)	<0.001
WHR	0.80 ± 0.07	0.91 ± 0.06	<0.001
WHtR	0.44 ± 0.05	0.58 ± 0.06	<0.001
SBP	109.3 ± 11.5	122.3 ± 12.4	<0.001
DBP	67.0 ± 9.8	75.2 ± 7.9	<0.001
hsCRP	0.32 (0.15–1.00)	2.11 (1.00–3.19)	<0.001
Triglyceride	69.0 (50.0–90.0)	97.5 (68.3–133.3)	<0.001
Total cholesterol	168.7 ± 32.7	167.4 ± 25.9	0.817
HDL	54.0 (48.0–61.0)	43.0 (38.0–51.0)	<0.001
LDL	91.8 ± 26.4	100.5 ± 21.5	0.053
HbA1c^b^	5.4 (5.2–5.5)	5.4 (5.2–5.6)	0.083
UA	5.4 ± 1.2	7.0 ± 1.5	<0.001
AST	20.0 (18.0–24.0)	30.5 (23.0–41.0)	<0.001
ALT	12.0 (10.0–17.0)	44.0 (25.8–64.0)	<0.001
γ-GT^c^	15.0 (13.0–19.0)	26.0 (19.0–42.5)	<0.001
HOMA-IR	1.1 (0.9–1.3)	2.1 (1.8–2.7)	<0.001
TNF-α	3.1 (0.8–7.9)	1.2 (0.0–7.0)	0.065
IL-6	1.9 (0.5–3.9)	2.8 (0.3–6.2)	0.117
Adiponectin	32.7 (21.4–51.9)	19.7 (14.1–25.1)	<0.001
M30	63.5 (45.2–85.0)	175.4 (114.0–318.4)	<0.001
US-FLI score	0.0 (0.0–0.0)	6.0 (3.0–8.0)	<0.001

*Statistics include percentage (%), mean (SD), or median (25th and 75th percentiles)*.

a *The definition of non-obesity and obesity was based on criteria reported by Chen et al.(13)*.

b *One missing data point of HbA1c in non-obesity and obesity group, respectively*.

c *One missing data point of γ-GT in obesity group*.

*BH, body height; BW, body weight; BMI, body mass index; WHR, waist to hip ratio; WHtR, waist to height ratio; SBP, systolic blood pressure; DBP, diastolic blood pressure; hsCRP, high sensitivity C-reactive protein; HDL, High-density lipoprotein; LDL, low-density lipoprotein; UA, uric acid; AST, aspartate aminotransferase; ALT, alanine aminotransferase; γ-GT, γ-glutamyltransferase; HOMA-IR, homeostatic model assessment insulin resistance; TNF, Tumor necrosis factor; IL-6, interleukin 6; M30, caspase-cleaved cytokeratin 18 fragment; US-FLI, ultrasonographic fatty liver indicator*.

### Semi-quantitative US-FLI Score Is Markedly Correlated With the Weight-To-Hip Ratio and Weight-To-Height Ratio

US-FLI score that ranges from 2 to 8 was significantly higher in the obesity group than that in the non-obesity group (0.00 [0.00–0.00] vs. 6.00 [3.00-8.00], respectively; *p* < 0.001) (Table 1). In the non-obesity group, hsCRP, triglyceride, ALT, γ-GT, TNF-α, and M30 were significantly associated with US-FLI score using simple linear regression (all *p* < 0.05) (Table 2). In the obesity group, the BW, BMI, WHR, WHtR, blood pressure (systolic and diastolic), HDL, uric acid, AST, ALT, γ-GT, adiponectin, and M30 were significantly associated with US-FLI score using simple linear regression (all *p* < 0.05) (Table 2). Specifically, the regression coefficient associated with WHR was 23.26, suggesting that each 0.1-unit increase in WHR is associated with a 2.326-unit increase in US-FLI score. The regression coefficient associated with WHtR was 23.39, suggesting that each 0.1-unit increase in WHtR is associated with a 2.339-unit increase in US-FLI score.

**Table 2:** Factors associated with semi-quantitative ultrasonographic fatty liver indicator (US-FLI) score analyzed using simple linear regression.

	**US-FLI score**					
	**Non-obesity^a^ (*n* = 59)**			**Obesity^a^ (*n* = 58)**		
**Variables**	**β (95% CI)**	**Constant**	***P***	**β (95% CI)**	**Constant**	***P***
Age	−0.04 (−0.21 to 0.13)	1.22	0.643	0.17 (−0.17 to 0.50)	2.84	0.322
BW	−0.00 (−0.04 to 0.03)	0.90	0.826	0.05 (0.01 to 0.10)	0.93	0.014
BH	−0.01 (−0.04 to 0.02)	2.72	0.411	0.03 (−0.04 to 0.10)	0.47	0.426
BMI	0.07 (−0.09 to 0.23)	−0.73	0.370	0.27 (0.10 to 0.44)	−2.88	0.003
WHR	3.80 (−1.84 to 9.44)	−2.33	0.182	23.26 (11.60 to 34.92)	−16.07	<0.001
WHtR	6.85 (−0.40 to 14.09)	−2.32	0.063	23.39 (11.90 to 34.87)	−8.51	<0.001
SBP	0.00 (−0.03 to 0.03)	0.63	0.972	0.08 (0.01 to 0.14)	−4.30	0.018
DBP	0.01 (−0.03 to 0.05)	0.14	0.676	0.14 (0.04 to 0.23)	−5.13	0.007
hsCRP	0.39 (0.01 to 0.77)	0.43	0.045	0.11 (−0.15 to 0.35)	4.72	0.405
Triglyceride	0.01 (0.00 to 0.02)	−0.10	0.027	0.01 (−0.01 to 0.02)	4.38	0.341
Total cholesterol	0.00 (−0.01 to 0.02)	0.24	0.648	−0.01 (-0.04 to 0.03)	5.79	0.774
HDL	−0.01 (−0.05 to 0.02)	1.34	0.519	−0.12 (−0.21 to −0.03)	10.30	0.012
LDL	0.01 (−0.01 to 0.02)	−0.08	0.247	0.02 (−0.02 to 0.06)	2.83	0.245
HbA1c	−0.13 (−0.67 to 0.40)	1.43	0.623	−0.10 (−1.08 to 0.88)	5.70	0.832
UA	0.21 (−0.10 to 0.52)	−0.44	0.182	0.84 (0.33 to 1.35)	−0.83	0.002
AST	0.03 (−0.01 to 0.08)	−0.06	0.149	0.04 (0.01 to 0.07)	3.68	0.014
ALT	0.03 (0.01 to 0.06)	0.15	0.017	0.04 (0.02 to 0.06)	2.85	<0.001
γ-GT	0.05 (0.01 to 0.08)	−0.14	0.01	0.08 (0.03 to 0.12)	2.60	0.001
HOMA-IR	0.80 (−0.09 to 1.69)	−0.28	0.076	0.37 (−0.13 to 0.88)	4.11	0.145
TNF-α	0.03 (0.01 to 0.05)	0.47	0.008	0.03 (−0.06 to 0.12)	4.87	0.492
IL-6	0.00 (−0.04 to 0.03)	0.71	0.806	0.00 (−0.02 to 0.03)	4.99	0.732
Adiponectin	−0.01 (−0.03 to 0.01)	1.12	0.214	−0.1 (−0.15 to −0.04)	7.24	0.001
M30	0.01 (0.00 to 0.02)	−0.10	0.01	0.01 (0.00 to 0.01)	3.86	0.003

a *The definitions for non-obesity and obesity were based on criteria reported by Chen et al. (13)*.

*CI, confidence interval; BW, body weight; BH, body height; BMI, body mass index; WHR, waist to hip ratio; WHtR, waist to height ratio; SBP, systolic blood pressure; DBP, diastolic blood pressure; hsCRP, high sensitivity C-reactive protein; HDL, High-density lipoprotein; LDL, low-density lipoprotein; UA, uric acid; AST, aspartate aminotransferase; ALT, alanine aminotransferase; γ-GT, γ-glutamyl transferase; HOMA-IR, homeostatic model assessment insulin resistance; TNF, tumor necrosis factor; IL-6, interleukin 6; M30, caspase-cleaved cytokeratin 18 fragment*.

### Multiple Linear Regression for Factors Associated With US-FLI Score

In the non-obesity group, the sample correlation coefficients between M30 and ALT and γ-GT were 0.58 and 0.51, respectively (all *p* < 0.05). In the obesity group, the sample correlation coefficients between WHtR and BW, BMI and WHR were 0.29, 0.68, 0.74, respectively (all *p* < 0.05). The sample correlation coefficient between M30 and AST, ALT, and γ-GT were 0.85, 0.79, and 0.64, respectively (both *p* < 0.05). The sample correlation coefficient between SBP and DBP were 0.48 (*p* < 0.05). In Table 3, after excluding the highly homogenous and markedly correlated variables (i.e., ALT and γ-GT in non-obesity group; BW, BMI, WHR, AST, ALT, γ-GT, and systolic blood pressure in obesity group), we adjusted for potential confounding factors (i.e., WHtR, hsCRP, triglyceride, TNF-α, and M30 in the non-obesity group and WHtR, DBP, uric acid, HDL, adiponectin, and M30 in the obesity group); see Table 3. TNF-α and M30 were significantly associated with US-FLI score in the non-obesity group (both *p* < 0.05). WHtR, uric acid, adiponectin, and M30 were significantly associated with US-FLI score in the obesity group (all *p* < 0.05). Specifically, each 0.1-unit increase in WHtR was associated with a 1.896-unit increase in US-FLI score in the multiple linear regression (regression coefficient: 18.96, 95% CI: 9.71–28.20) after adjusting for uric acid, adiponectin, and M30.

**Table 3:** Factors associated with semi-quantitative ultrasonographic fatty liver indicator (US-FLI) score analyzed using multiple linear regression.

**US-FLI score**							
**Non-obesity^a^ (*n* = 59)**				**Obesity^a^ (*n* = 58)**			
**Variables**	**β (95% CI)**	**Constant**	***P***	**Variables**	**β (95% CI)**	**Constant**	***P***
TNF-α	0.03 (0.01 to 0.05)	−0.42	0.002	WHtR	18.96 (9.71 to 28.20)	−10.64	<0.001
M30	0.01 (0.00 to 0.02)		0.003	UA	0.77 (0.38 to 1.15)		<0.001
				Adiponectin	−0.06 (−0.10 to −0.02)		0.004
				M30	0.003 (0.001 to 0.006)		0.010

a *The definition of non-obesity and obesity was based on the criteria reported by Chen et al. (13)*.

*CI, confidence interval; M30, caspase-cleaved cytokeratin 18 fragment; TNF, tumor necrosis factor; UA, uric acid; WHtR, waist to height ratio*.

### Diagnostic Value of US-FLI Score to Predict NAFLD With Hepatitis

**Figure 1:**
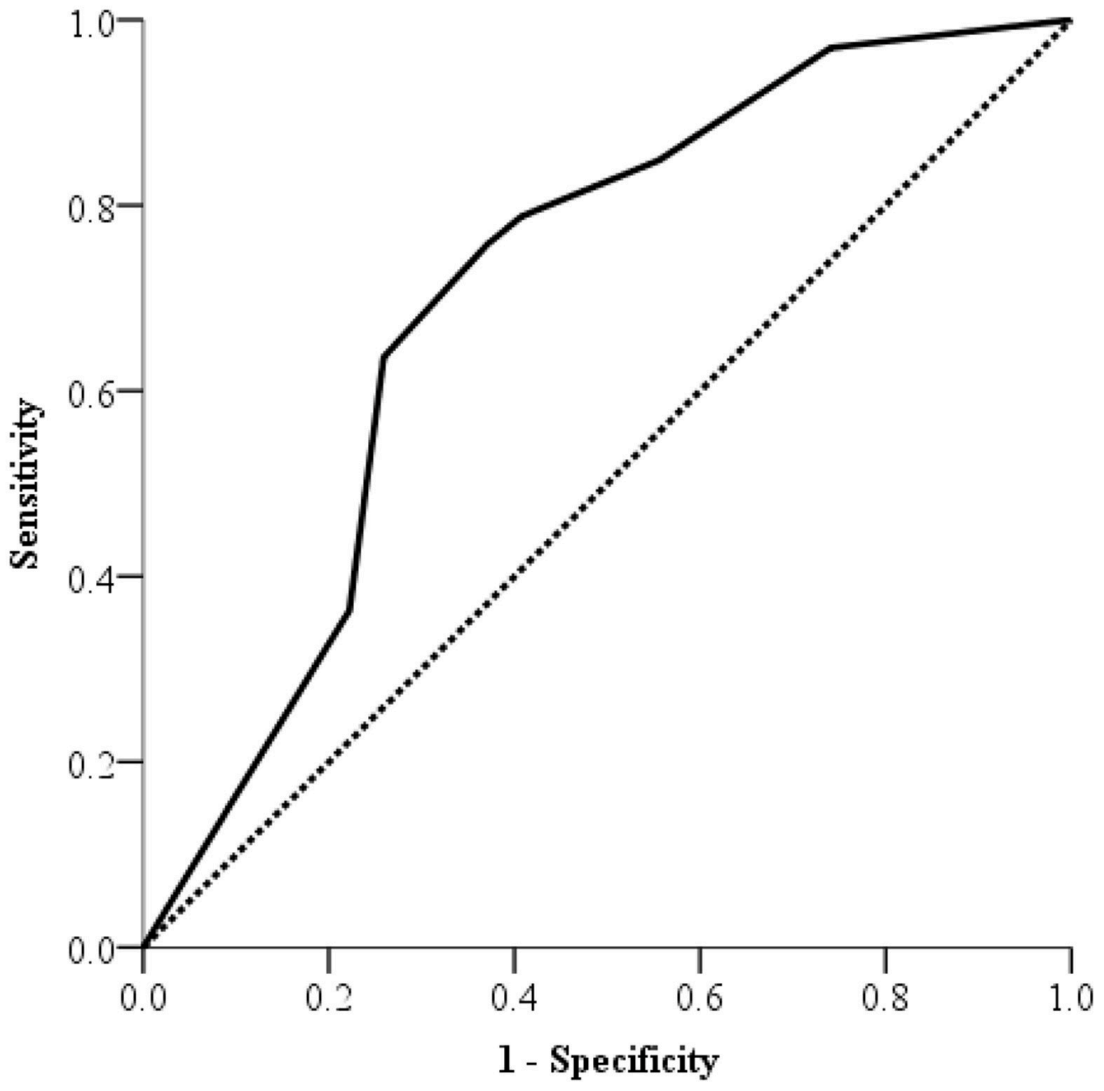
Receiver operating characteristic (ROC) curve of US-FLI scores for predicting hepatitis in non-alcoholic fatty liver disease in children.

Among 117 participants, 33 (28.2%) had NAFLD with hepatitis and 27 (23.1%) NAFLD without hepatitis. Among 60 patients with NAFLD, the ROC curve of semi-quantitative US-FLI score to predict hepatitis in children with NAFLD is shown in Figure 1. The area under the ROC curve was 0.71 (95% CI: 0.572–0.847; *p* = 0.005) to predict hepatitis in children with NAFLD. The best cut-off point of semi-quantitative US-FLI score to predict hepatitis in children with NAFLD was ≥6. Positive predictive value (PPV) was 71.4% with a sensitivity of 75.8% and a specificity of 63.0%.

## Discussion

NAFLD in children is strongly associated with cardiovascular dysfunction, prediabetes, and type 2 diabetes mellitus (15, 16). Public health awareness, early recognition, and adequate intervention (e.g., healthy diet, lifestyle modifications, and exercise, etc.) are urgently needed to reduce the burden of disease in the community.

The general indication of liver biopsy in children for NAFLD is to exclude other treatable or advanced liver disease, before medical or surgical treatment, and for clinical research (17). However, because parental concerns are a major hindrance to such an invasive procedure, liver biopsy should be considered to evaluate NAFLD after the evaluation of biochemical or non-invasive imaging facilities.

Abdominal USG is a convenient and cost-effective imaging modality to evaluate patients with NAFLD. Formerly, NAFLD was diagnosed using USG graded as follows: mild, moderate and severe (18). However, this grading system is subjective and may vary with different operators. Semi-quantitative US-FLI score, which was proposed by Ballestri et al. (11), had been shown to have a high correlation with the severity of NAFLD histopathology in adults, and be more objective with different operators. To our knowledge, few studies have evaluated the correlation between obesity, US-FLI score, and the associated parameters of NAFLD in children.

BMI is not an accurate marker for adiposity measures because it is difficult to distinguishing bone and muscle from adipose tissue. Central obesity, instead of BMI, has been reported to correlate better with clinical outcomes than BMI (19). Among the parameters (e.g., waist circumference, WHR, or WHtR) that are associated with central obesity, WHtR is considered to be a more powerful predictor of NAFLD in children and adolescents (20). We used WHtR as a predictor in multiple linear regression model, and the results suggest that WHtR is highly correlated with US-FLI score in children with obesity. Furthermore, some non-invasive serum biomarkers or inflammatory markers have been used to predict NAFLD severity (4, 6–9, 20). Adiponectin, which is the most abundant and white adipose tissue-specific adipokine, is significantly linked to NAFLD, IR, and metabolic syndrome (21–23). The level of adiponectin that decreased while increasing fat mass was highly correlated with steatosis grade and severity of NAFLD (22). In addition, M30, a marker from hepatocellular apoptosis, has also shown to be a promising biomarker for children with NAFLD. In children, M30 was highly associated with NASH and predictor of NASH on liver biopsy (9). Additionally, uric acid is a well-studied independent risk factor for NAFLD and independently associated with NASH in pediatric patients (24). In this study, these results indicate that US-FLI score was strongly correlated with WHtR, uric acid, adiponectin, and M30 levels in children with obesity. Besides, US-FLI score was significantly correlated with TNF-α and M30 levels among children without obesity in this study. Researchers previously reported that TNF-α might play an important role in the development and progression of NAFLD (25), and this might explain the correlation between US-FLI score and TNF-α. Therefore, US-FLI score has potential to be developed as a useful non-invasive tool to estimate NAFLD in pediatric population.

Furthermore, the result of ROC in this study also suggest that US-FLI score can distinguish NAFLD with from without hepatitis in children. The PPV of 71.4% suggests that children with NAFLD and US-FLI score ≥6 are at relatively high risk for hepatitis. In practice, physicians could use the US-FLI score to suggest further blood sampling, intensive intervention, or liver biopsy arrangement in cases where US-FLI score is ≥6. Hence, this convenient US-FLI score could be used as a first-line non-invasive screen tool for hepatitis in the children with NAFLD.

We acknowledge that our study has limitations. Although liver biopsy remains the gold standard for diagnosing patients with NASH and assessing severity, the procedure was not performed in our study because of parental concerns about complications. Therefore, we could not compare US-FLI scores and liver biopsy findings. Another limitation is the cross-sectional design of our study. Future cohort studies on US-FLI scores in children with NAFLD is warranted.

Altogether, we found that the WHtR, uric acid, adiponectin, and M30 levels strongly correlated with the US-FLI score in the children with obesity. A cut-off US-FLI score of ≥ 6 can also predict hepatitis in children with NAFLD. Hence, the US-FLI score is a useful tool for estimating NAFLD.

## Author Contributions

H-KL, M-CY, and C-CT: study design; H-KL, M-CY, Y-TS, and C-CT: study execution; H-KL, C-MT, Y-FW, I-CL, M-CY, and C-CT: data analysis and interpretation; H-KL, M-CY, and C-CT: manuscript writing/editing. All authors of this manuscript have read manuscript and given final approval of the version to be published.

### Conflict of Interest Statement

The authors declare that the research was conducted in the absence of any commercial or financial relationships that could be construed as a potential conflict of interest.

## Abbreviations

NAFLD: Non-alcoholic fatty liver disease
AST: aspartate aminotransferase
ALT: alanine aminotransferase
γ-GT: γ-glutamyltranspeptidase
IR: insulin resistance
TNF-α: tumor necrosis factor-alpha
IL-6: interleukin-6
NASH: non-alcoholic steatohepatitis
USG: ultrasonography
BMI: body mass index
WHR: waist to hip ratio
WHtR: waist to height ratio
ALK-P: alkaline phosphatase
TC: total cholesterol
TG: triglyceride
HDL: high density lipoprotein
LDL: low-density lipoprotein
HbA1c: glycated hemoglobin.
